# Edge-Enabled Real-Time Gait Assessment for Degenerative Spinal Disease Using Wearable Inertial Sensors

**DOI:** 10.3390/s26144339

**Published:** 2026-07-08

**Authors:** Kuei-Ann Wen, Li-Hsieh Lin, Jiun-Lin Yan, Chen-Nen Chang, David Shih

**Affiliations:** 1 Department of Electronics Engineering, National Yang Ming Chiao Tung University, Hsinchu City 30010, Taiwan; twtstella@gmail.com (K.-A.W.); david0409118@gmail.com (D.S.); 2 Department of Neurosurgery, Chang Gung Memorial Hospital, Keelung City 20401, Taiwan; color_genie@hotmail.com; 3 Department of Neurosurgery, Chang Gung Memorial Hospital, Taoyuan City 333423, Taiwan; chennen.chang@gmail.com

**Keywords:** gait analysis, degenerative spinal disease, inertial measurement unit

## Abstract

Gait analysis is used in the diagnosis, rehabilitation, and longitudinal monitoring of degenerative spinal disease (DSD). However, conventional gait assessment commonly depends on subjective visual observation or laboratory-based motion-capture systems, which restrict accessibility and routine clinical use. This study presents an edge-enabled real-time gait analysis framework for DSD using two ankle-worn inertial measurement units (IMUs). The proposed framework integrates causal gait-event detection with spatiotemporal gait estimation, including stride length, stride height, stride frequency, and swing ratio, across Regular-, Toe-, Heel-, and Tandem-Walk tasks. To improve the stability of wearable gait estimation, the framework incorporates a cycle-wise initial sensor-orientation correction strategy with inter-cycle horizontal velocity continuity, reducing reliance on conventional zero-velocity update (ZUPT) resetting. A percentile-referenced, separability-weighted composite score was also developed to combine average gait performance, step-to-step variability, and gait asymmetry into an interpretable clinical index. Algorithm validation was conducted using an optical motion-capture system as the reference standard. The proposed framework, however, is intended for deployment using ankle-worn IMUs and edge-based computation without requiring optical cameras, reflective markers, or dedicated motion-capture laboratories during routine operation. Experimental results showed centimeter-level spatial estimation accuracy. The composite scoring framework achieved accuracy/F1-scores of 0.971/0.981 for Regular-Walk, 0.934/0.956 for Toe-Walk, 0.955/0.970 for Heel-Walk, and 0.876/0.919 for Tandem-Walk. Feature analysis indicated that stride length, stride frequency, swing ratio, and step-to-step variability provided the greatest discrimination between healthy controls and spinal patients, with stronger group separation observed in Regular-, Toe-, and Heel-Walk tasks. These results suggest that ankle-mounted IMU sensing combined with lightweight edge-based computation and interpretable gait scoring may provide a practical approach for point-of-care gait assessment and remote functional monitoring in DSD. The proposed system operates using only two ankle-mounted IMUs during routine deployment, while optical motion capture was employed exclusively as a laboratory reference for validation.

## 1. Introduction

Gait is a fundamental motor activity that emerges from coordinated interactions among the nervous, musculoskeletal, and cardiorespiratory systems [1]. The central nervous system continuously integrates visual, tactile, proprioceptive, and vestibular inputs and issues motor commands executed by the musculoskeletal system. Accordingly, quantitative gait analysis has become a practical biomarker for neurological, musculoskeletal, and spinal disorders—including Parkinson’s disease (PD), Huntington’s disease (HD), cerebral palsy (CP), multiple sclerosis (MS), and osteoarthritis (OA)—because spatiotemporal parameters such as velocity, stride length, and cadence are sensitive to disease onset and progression [1,2].

Degenerative lumbar spine disease (DSD) imposes a substantial worldwide burden. Population-level estimates indicate that low back pain and degenerative lumbar conditions affect hundreds of millions of individuals, leading to marked disability and healthcare utilization [3]. Clinically, patients with lumbar spinal stenosis, disk herniation, or chronic low back pain often exhibit slower walking speed, shortened steps, prolonged double support, and heightened instability; these alterations degrade function and quality of life and complicate rehabilitation planning [1,4]. Consequently, reliable and scalable tools that quantify gait impairment in DSD are of direct clinical relevance for screening, triage, and longitudinal monitoring. Recent investigations have further shown that gait performance in chronic low back pain is associated not only with biomechanical dysfunction but also with pain intensity, executive function, psychosocial factors, and cortical activity, highlighting the multidimensional nature of gait impairment in spinal disorders [5].

Historically, laboratory instrumentation—electronic walkways and optical motion capture—has served as the reference standard for precise gait quantification. While these systems deliver high-fidelity kinematics and event timing, they require dedicated facilities, trained personnel, and controlled environments that limit throughput and broader deployment [4]. In parallel, wearable sensing has matured rapidly. Recent reviews document expanding use of IMUs to estimate spatiotemporal parameters, detect gait events, and characterize variability in free-living settings, with growing attention to drift mitigation, zero-velocity update (ZUPT) alternatives, and model-based estimation [2]. Wearable, edge-computing approaches therefore offer a promising pathway to bridge laboratory-grade accuracy with practical reach. Recent developments in embedded biomedical sensing systems have further emphasized the importance of hardware–software co-design, low-power acquisition architectures, and real-time signal processing for practical healthcare deployment. Beyond algorithmic performance, successful clinical translation increasingly depends on efficient integration between sensing hardware, embedded computation, and application-oriented monitoring frameworks. At the same time, wearable and contactless monitoring technologies are evolving toward continuous, multi-modal, and multi-task assessment paradigms that support long-term health monitoring outside laboratory environments.

From a disease-specific perspective, comparative analyses across DSD subtypes highlight both shared and task-dependent gait signatures [4]. Patients frequently demonstrate reduced stride length and cadence, together with increased asymmetry and step-to-step variability; however, effect sizes depend on pathology and walking condition (e.g., self-selected versus constrained tasks). These observations motivate two design principles for sensor-based DSD assessment: (i) protocols should probe multiple walking tasks to expose complementary biomechanical demands, and (ii) feature sets should extend beyond means to include variability and asymmetry metrics that capture instability and compensatory strategies [4].

Motivated by these needs, this study develops an edge-enabled, real-time gait analysis framework tailored to spinal disorders. Building on advances in wearable sensing and algorithmic estimation [2], the system maps sensor-frame signals to Earth coordinates, performs robust event detection, and yields clinically interpretable spatiotemporal parameters—stride length, stride height, stride frequency, and swing ratio. To capture pathology-relevant instability, the protocol spans four walking tasks and augments conventional parameters with variability and asymmetry features. For decision support, we aggregate features into a percentile-normalized, separability-weighted composite score that transparently stratifies healthy controls and spinal patients. By validating against motion-capture ground truth and demonstrating on-device operation, this work aims to narrow the gap between laboratory-grade metrics and scalable, point-of-care assessment in degenerative spinal disease [3,4].

A growing body of work has quantified gait alterations in spinal disorders using laboratory instrumentation [5,6,7,8,9,10,11,12,13,14]. Across diagnoses such as chronic low back pain, lumbar disk herniation (LDH), and lumbar spinal stenosis (LSS), studies consistently report slower walking speed, shorter steps, prolonged double support, and increased instability. For example, Bonab et al. analyzed 50 patients and showed that higher pain intensity was negatively correlated with step/stride length, cadence, and velocity [12]. Lee et al. demonstrated that patients with LDH exhibit significantly greater gait variability than healthy individuals, indicating compromised stability [8]. In a 30 m overground task, Perring et al. found large LSS–control separations in cadence (−14%), step length (−24%), walking velocity (−37%), and step duration (+16%) [11]. While electronic walkways and optical motion capture provide high-fidelity spatiotemporal and kinematic measurements and are widely regarded as reference standards, they require dedicated facilities, expert operators, and controlled environments, constraining throughput and limiting utility for routine follow-up or home/community monitoring [5,6,7,8,9,10,11,12,13,14].

In contrast, wearable IMUs offer low cost, small form factor, and straightforward deployment, enabling ambulatory assessment and telemedicine workflows. Recent reviews and application studies document robust IMU pipelines for estimating spatiotemporal parameters, detecting gait events, and characterizing variability in real-world contexts, including Parkinson’s disease (PD) [15,16,17,18,19,20,21,22], stroke [20,21,22,23], and other neurological conditions [24,25]. Recent validation studies have further demonstrated the feasibility of IMU-based systems for estimating clinically relevant spatiotemporal gait parameters in neurological populations, supporting their broader application in wearable gait assessment and longitudinal monitoring [17]. These works increasingly emphasize feature selection and composite indices, alongside algorithmic refinements for drift mitigation and model-based estimation, narrowing the gap with laboratory systems while expanding ecological validity. More recently, intelligent biomedical sensing systems have incorporated adaptive filtering, model-based estimation, sensor-fusion strategies, and lightweight machine-learning techniques to improve robustness under noisy and dynamic operating conditions [2]. These developments highlight the growing need to balance sensing performance, computational efficiency, and clinical interpretability in wearable healthcare applications. Recent studies have also demonstrated the feasibility of IMU-based systems for biomechanical characterization, balance assessment, and clinical gait evaluation beyond conventional spatiotemporal analysis [22]. Nevertheless, IMU studies tailored specifically to degenerative lumbar pathology remain comparatively sparse, often focusing on single walking conditions or mean values rather than task-dependent impairments, step-to-step variability, and asymmetry. This landscape motivates edge-enabled, real-time IMU frameworks for spinal disease that (i) probe multiple gait tasks to reveal complementary biomechanical demands and (ii) integrate discriminative variability/asymmetry descriptors into interpretable composite scores—thereby extending laboratory-grade insights to scalable, point-of-care assessment.

In this study, to address the limitations of prior lab-bound or mean-only approaches, this work delivers an edge-enabled, real-time gait analysis framework expressly tailored to degenerative spinal disease. Participants comprising healthy controls and individuals with spinal pathology were recruited from clinical settings, and four complementary walking tasks—Regular-, Toe-, Heel-, and Tandem-Walk—were conducted to elicit task-dependent impairments with clear clinical interpretability. These tasks are routinely adopted in neurological and musculoskeletal assessments, enabling the proposed framework to capture functionally relevant gait alterations under realistic clinical testing conditions. On-device, two ankle-worn IMUs feed an algorithm that maps sensor-frame signals to Earth coordinates, performs finite-state-machine gait-event detection, and outputs stride length, stride height, stride frequency, and swing ratio in real time, while robustness beyond ZUPT is achieved via cycle-start horizontal velocity initialization and a dynamic initial-orientation self-calibration that enforces inter-cycle velocity continuity. Moving beyond mean summaries, we derive, for each parameter, step-wise average, variation, and variation ratio for the left, right, and both feet (36 features per task), which mitigates anthropometric confounding and sensitively captures gait variability and asymmetry—two hallmarks of spinal pathology. Using this representation, we identify the parameter–task pairs that maximize group separability—namely stride length (SL), stride frequency (SF), and swing ratio (SR) in Regular- and Toe-Walk and SL in Heel-Walk—while also confirming the limited discriminative capacity of Tandem-Walk, consistent with its constrained step geometry. For decision support, all features are mapped to healthy-based percentiles and weighted by between-group z-scores to form a transparent composite score suitable for participant-level classification and longitudinal tracking. Accuracy is validated against motion-capture ground truth, and a mobile implementation demonstrates real-time operation via Bluetooth streaming, thereby narrowing the gap between laboratory-grade metrics and scalable, point-of-care assessment.

## 2. Materials and Methods

### 2.1. Sensors and Placement

A two-sensor wearable platform (Rabboni IMUs, SIPP Technology Corporation, Hsinchu City, Taiwan), InvenSense ICM-20689 implemented to acquire tri-axial accelerations and tri-axial angular velocities from the left and right ankles. Each unit was configured to ±8 g (accelerometer) and ±1000 deg/s (gyroscope) and sampled at 50 Hz, based on the Nyquist theorem for gait harmonics while preserving battery life and Bluetooth throughput for edge (on-device) processing. Previous studies have shown that moderate sampling frequencies are sufficient for ambulatory gait and activity monitoring using body-worn inertial sensors [26,27]. Therefore, the proposed framework prioritized stable real-time operation and low computational burden rather than excessively high sampling rates. The ankle-worn IMUs were attached to the distal shank immediately proximal to the superior borders of the medial and lateral malleolus, which served as reproducible palpable bony landmarks. The sensors were oriented such that their mediolateral axes were aligned as consistently as possible with the anatomical ankle joint axis approximated by the line connecting the medial and lateral malleolus. This ankle-level placement was selected because distal-shank signals exhibit pronounced angular excursions and clear heel-strike and toe-off signatures, thereby improving gait-phase prediction and stride-level parameter estimation [28].

**Figure 1:**
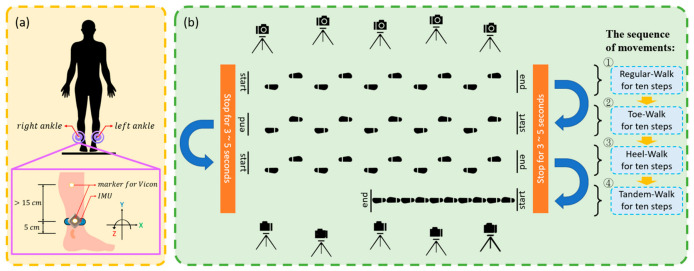
(**a**) The IMUs were attached to the distal shank immediately proximal to the medial and lateral malleolus on both lower limbs. Reflective Vicon markers were positioned at the center of each ankle-mounted IMU and on the proximal shank with sufficient separation from the IMU to minimize optical occlusion and mechanical interference during walking trials. (**b**) The laboratory was equipped with a Vicon motion-capture system for algorithm validation while participants performed the predefined walking tasks with synchronized IMU and Vicon recording.

At the commencement of each trial, the two IMUs were time-aligned to the mobile host via a broadcast trigger, and any residual offset was removed by timestamp correction; data quality was monitored online, and segments exhibiting >100 ms packet dropouts or visible strap slippage were repeated. Raw counts were converted to SI units using factory calibration constants, and per-session biases (gyroscope offset and accelerometer zero-g offset) were estimated from an initial quiet-stance period and removed. For algorithm validation, a camera-based Vicon VERO system (Vicon Motion Systems Ltd., Oxford, UK) provided motion-capture ground truth. The Vicon system was used solely as a reference standard for validating the proposed gait-estimation algorithm and was not involved in gait-event detection, parameter estimation, score generation, or operation of the mobile application. All real-time gait parameters reported by the proposed framework were generated exclusively from IMU measurements. Accordingly, optical cameras are required only during the laboratory validation phase and are not required for routine clinical deployment of the proposed system. Two reflective markers were affixed to each lower limb—one co-located with the ankle-mounted IMU on the distal shank and one positioned on the proximal shank with sufficient separation from the IMU to reduce optical occlusion and mechanical interference during walking trials (Figure 1). Marker trajectories were used to reconstruct the lower-limb position, velocity, and acceleration, while lower-leg angular velocity was derived from relative marker vectors for gait-event identification and stride-level spatial parameter validation.

This configuration is consistent with prior evidence that compact sensing with modest sampling can yield reliable activity/posture recognition and speed/incline estimation when placement and signal conditioning are carefully controlled [26,27]; in our case, the ankle-mounted 50 Hz setup provides sufficient temporal resolution for phase-critical events while remaining computationally light for edge execution and suitable for long-duration ambulatory recordings in clinical corridors, thereby balancing accuracy, robustness, and deployability for the real-time algorithms described next.

### 2.2. Arrangements for Performance Validation

Three cohorts were enrolled in this study for algorithm validation and clinical gait assessment. The distribution of recorded gait trials for each walking task the demographic, anthropometric, and clinical characteristics of all participants are presented in Table 1.

Group I consisted of 15 healthy adults and was used exclusively for algorithm validation under laboratory conditions. Participants wore two ankle-mounted inertial measurement units (IMUs) together with four reflective markers while performing all walking tasks in a motion-analysis laboratory instrumented with a Vicon optical motion-capture system. The synchronized Vicon recordings served as the reference standard for validating gait-event detection and spatial–temporal gait parameter estimation.

Group II comprised 37 healthy adults who served as the healthy control (HC) reference cohort for clinical gait comparison and classification analysis. Participants wore two ankle-mounted IMUs and performed the same walking tasks along a straight indoor corridor under supervised experimental conditions.

Group III comprised 101 patients with degenerative spinal disorders recruited at Chang Gung Memorial Hospital, Keelung, between April 2021 and August 2023 (Chang Gung Medical Foundation IRB No. 202001203B0). Participants in this cohort wore two ankle-mounted IMUs and completed the same gait assessment protocol in a clinical environment. Diagnoses included low back pain, lumbar disk herniation, and lumbar spinal stenosis, with some patients presenting multiple concurrent spinal-related conditions.

The spinal patient cohort represented heterogeneous clinical conditions associated with locomotor dysfunction commonly encountered in neurological and orthopedic practice. Clinical manifestations included low back pain, sciatica, neurogenic claudication, lower-extremity weakness, sensory abnormalities (e.g., numbness), and balance instability. Because the present study focused on functional gait impairment under realistic clinical conditions rather than disease-specific pathology, patient severity was characterized using symptom-oriented clinical assessment rather than a unified disease-specific staging system.

All participants completed four complementary walking tasks, including Regular-Walk, Toe-Walk, Heel-Walk, and Tandem-Walk. Each recording sequence typically contained approximately 6–10 consecutive gait cycles, depending on participant walking speed and task completion. The resulting dataset enabled evaluation of both algorithmic performance and clinically relevant gait characteristics across healthy and spinal-pathology populations.

### 2.3. Experimental Protocol

As illustrated in Figure 1, each participant completed four walking tasks in the following sequence: (i) Regular-Walk at a self-selected pace (10 steps), (ii) Toe-Walk (10 steps), (iii) Heel-Walk (10 steps), and (iv) Tandem-Walk with heel-to-toe contact (10 steps). In the Vicon laboratory, participants turned at the end of each bout due to space constraints and paused for 3–5 s at the start and end of each task; these turn/pausing intervals were excluded from all subsequent analyses.

### 2.4. IMU Signal Processing

To condition the raw gyroscope signals for downstream event detection and spatial estimation, we applied a second-order Butterworth low-pass filter with a 12 Hz cutoff to the ankle angular velocities. This choice follows established practice that low-pass filtering attenuates high-frequency noise, small involuntary tremors, and shoe–floor friction artifacts without distorting the gait waveform of interest [20]. Because fundamental gait frequency typically lies below ~2 Hz in level walking [29] and prior methodological work recommends a minimum sampling rate of ~35 Hz with LPF cutoffs > 10 Hz for lower-limb angular-velocity acquisition and conditioning [30], our 50 Hz sampling with a 12 Hz cutoff provides a conservative passband for preserving stride-scale dynamics while removing non-informative high-frequency content. Filtering was implemented causally for real-time operation, with per-trial state warm-up during the initial quiet-stance segment; identical settings were used for both limbs to ensure symmetry of processing.

### 2.5. Gait-Event Detection

**Figure 2:**
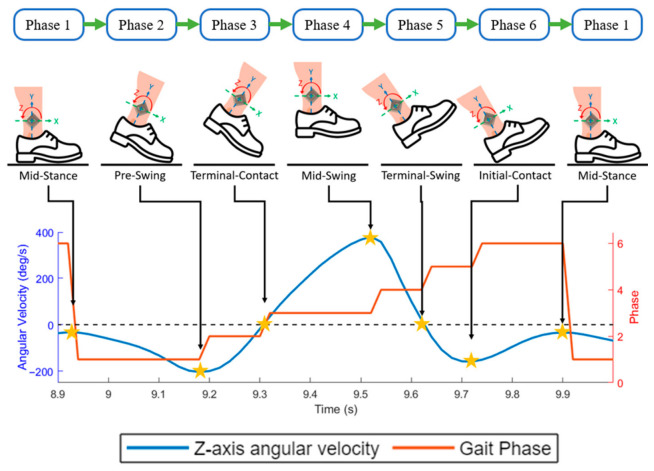
The six gait events and their patterns of ankle angular velocity in a gait cycle. The dotted horizontal line indicates the zero-reference level. Stars denote the locations of the six canonical gait phases on the waveform.

Human gait is a cyclic pattern [31]. We partitioned each gait cycle into six canonical phases and designed a finite-state machine (FSM) driven by ankle angular-velocity features (Figure 2): Mid-Stance (MSt), Pre-Swing (PS), Terminal-Contact (TC), Mid-Swing (MSw), Terminal-Swing (TSw), and Initial-Contact (IC) (Figure 2). For notational consistency, the left foot sagittal angular-velocity signal was sign-inverted so that right and left traces share the same polarity convention.

Mid-Stance (MSt). The foot bears full body weight; on the ankle sagittal gyroscope, this corresponds to the dominant peak within the negative-velocity region and is used to mark both cycle start and cycle end [18,23,30,32].

Pre-Swing (PS). The heel lifts while the toe remains in contact [33]; this aligns with the last valley in the negative angular-velocity region and is often termed heel-off (HO) [34].

Terminal-Contact (TC). The foot loses contact with the floor—also referred to as end-contact (EC) or toe-off (TO) [2,35]. Consistent with methodological studies, TC occurs near the zero-crossing preceding Mid-Stance rather than at the last negative valley [30,36]. Because Toe-Walk and Heel-Walk impose task-dependent forefoot or rear-foot constraints, we use the neutral term Terminal-Contact across all tasks (avoiding the task-specific “toe-off” misnomer in Heel-Walk).

Mid-Swing (MSw). The reference limb advances with maximal thigh flexion; on the gyroscope, this maps to the dominant peak in the positive-velocity region [30,37].

Terminal-Swing (TSw). The shank enters the final swing, preparing for ground contact; this corresponds to the first valley following Mid-Swing.

Initial-Contact (IC). The foot first touches the floor—termed heel-strike in level walking [2,16,32,34]. We adopt the task-agnostic label Initial-Contact, since heel-strike does not occur in Toe-Walk.

The FSM transitions are governed by these polarity- and extremum-based landmarks, with amplitude and duration guards to suppress spurious detections. This design aligns the event taxonomy with prior gyroscope-based mappings (particularly the zero-crossing proximity of TC/TO [30,36]) while remaining robust to task-induced waveform changes and inter-limb polarity.

### 2.6. Acceleration Transformation and Velocity Estimation

**Figure 3:**
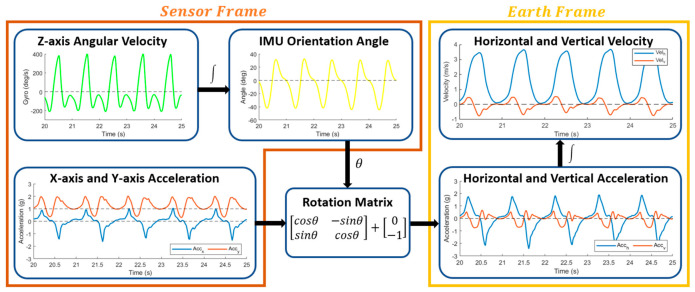
Transformation of accelerations from IMU coordinates to Earth coordinates and estimation of Earth coordinate velocities.

Since the three-axis accelerations and three-axis angular velocities recorded by the IMU are in the sensor coordinate system, it is necessary to transform them into the Earth coordinate system first [29], with the signal processing flow as shown in Figure 3.

Because participants are required to walk in a straight line for all gait movements during the experiment, we assume for simplicity that the sensors worn by the participants only move in the X-Y plane.

(1) $${Acc}_{z}\left(t\right)={Gyro}_{x}\left(t\right)={Gyro}_{y}\left(t\right)=0$$

Define θ as the angle between the gravity axis and the Y-axis in IMU frame. It is estimated through the integration of Z-axis angular velocity and reset at the start of each motion cycle that

(2) $$\theta \left(t\right)=\int_{t_{cycle\;start}}^{t}{Gyro}_{z}\left(\tau \right)d\tau +\theta \left(t_{cs}\right)$$

where $\theta (t_{cs})$ is the sensor orientation angle at the start of the gait cycle, and this will be discussed in the following section.

After obtaining the sensor orientation angle, it can be used to compute the sine and cosine functions, allowing us to estimate the horizontal and vertical acceleration in the Earth coordinate.

(3) $${Acc}_{h}\left(t\right)=\left(cos\theta \left(t\right){Acc}_{x}\left(t\right)+sin\theta \left(t\right){Acc}_{y}\left(t\right)\right)$$

(4) $${Acc}_{v}\left(t\right)=\left(sin\theta \left(t\right){Acc}_{x}\left(t\right)-cos\theta \left(t\right){Acc}_{y}\left(t\right)+1\right)$$

After obtaining the horizontal and vertical acceleration in Earth coordinates, we can calculate the horizontal and vertical velocity in Earth coordinates by integrating the acceleration.

(5) $${Vel}_{h}\left(t\right)=\int_{t_{cs}}^{t}{Acc}_{h}\left(\tau \right)d\tau +{Vel}_{h}\left(t_{cs}\right)$$

(6) $${Vel}_{v}\left(t\right)=\int_{t_{cs}}^{t}{Acc}_{v}\left(\tau \right)d\tau$$

To reduce spatial parameter drift, zero-velocity update (ZUPT) techniques have been widely adopted in mobile gait analysis systems [38,39]. Conventional ZUPT-based methods assume that the foot remains stationary during the Mid-Stance phase and therefore reset the estimated horizontal velocity to zero. However, synchronized Vicon measurements in our experiments consistently demonstrated residual forward ankle motion during Mid-Stance, indicating that strict zero-velocity assumptions may systematically underestimate stride length. To mitigate this effect, the cycle-start horizontal velocity was approximated using a simplified rotational kinematic model:

(7) $${Vel}_{h}\left(t_{cs}\right)=-{height}_{ankle}*{Gyro}_{Z}\left(t_{cs}\right)$$

where ${\;Gyro}_{Z}\left(t_{cs}\right)$ denotes the Z-axis angular velocity measured during the Mid-Stance event, and ${height}_{ankle}$ represents the vertical distance between the ankle-mounted sensor and the ground.

This formulation is based on the tangential velocity relationship v≈rω, where r represents the approximate distance between the foot–ground contact region and the ankle-mounted sensor, and ω denotes the angular velocity measured by the gyroscope. Under this simplified rotational kinematic approximation, the ankle-mounted sensor is assumed to undergo rotational motion around the foot–ground contact region during the Mid-Stance phase. Consequently, despite reduced foot motion, the ankle-mounted sensor may still retain a non-zero forward tangential velocity. Accordingly, replacing strict zero-velocity resetting with the estimated cycle-start velocity can mitigate stride-length underestimation commonly associated with conventional ZUPT-based approaches.

For implementation simplicity and to avoid subject-specific calibration, ${height}_{ankle}$ was approximated as a constant value of 15 cm in this study. Although this simplification does not fully account for inter-subject anthropometric variability, experimental validation against the Vicon motion-capture system demonstrated improved agreement with the ground-truth stride-length measurements compared with strict ZUPT-based velocity resetting.

### 2.7. Self-Correction of Initial Sensor Orientation Angle

To integrate angular velocity and recover the sensor’s orientation at each instant, an initial sensor orientation angle must be specified at the beginning of every gait cycle. Benoît Sijobert et al. [18] assumed that, at cycle onset, the sensor is aligned with the limb. Shenggao Zhu et al. [29] instead assumed a zero-motion instant and computed the initial angle by applying inverse trigonometric functions to the ratio of vertical to horizontal accelerations when the sensor is at this zero-motion instant. However, the initial orientation can vary across gait tasks and between individuals due to task-dependent posture and habitual walking patterns, so a fixed assumption may introduce bias that propagates through subsequent estimation.

**Figure 4:**
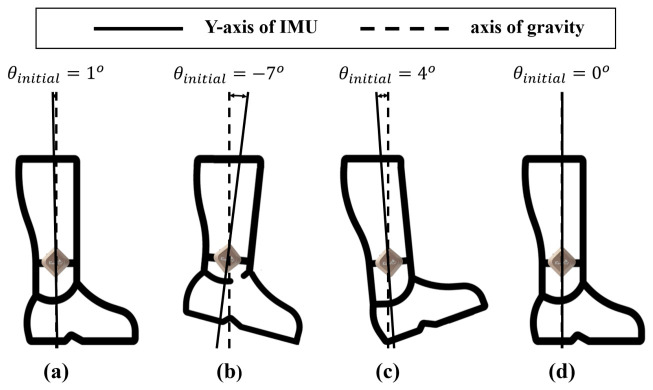
The initial sensor orientation angle at the Mid-Stance event. (**a**) Regular-Walk; (**b**) Toe-Walk; (**c**) Heel-Walk; (**d**) Tandem-Walk.

We also tried a range from −10 degrees to 10 degrees to find the most suitable initial sensor orientation angle for each of the four gait movements. Figure 4 shows the initial sensor orientation angles for Regular-Walk, Toe-Walk, Heel-Walk, and Tandem-Walk that result in the minimum mean absolute error of horizontal velocity at the end of each cycle. In addition to assigning different initial angles for different gait movements, we also propose a method for dynamically correcting the initial angles based on the error of horizontal velocity.

The horizontal velocity at the end of each gait cycle should be equal to the initial velocity at the start of the next gait cycle:

(8) $${\left(\int_{t_{cs}}^{t_{ce}}{Acc}_{h}\left(\tau \right)d\tau +{Vel}_{h}\left(t_{cs}\right)\right)}_{{step}_{i}}={\left({Vel}_{h}\left(t_{cs}\right)\right)}_{{step}_{i+1}}$$

**Figure 5:**
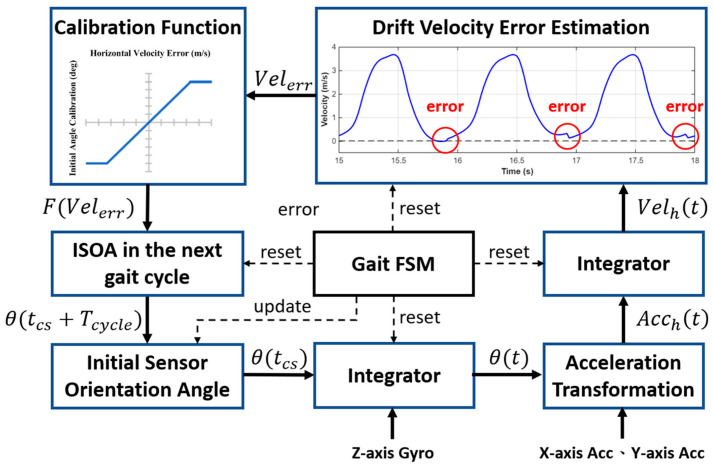
Dynamic correction of the initial sensor orientation angle based on horizontal velocity continuity between consecutive gait cycles. Solid arrows indicate the normal signal-processing flow and finite-state-machine (FSM) state transitions, whereas dashed arrows represent reset operations that may be triggered by the FSM from any state when error conditions are detected. Colors are used for visualization purposes only and do not convey additional scientific meaning.

However, due to errors in horizontal velocity, there exists a gap between the horizontal velocities at the end of one gait cycle and the start of the next, resulting in the horizontal velocity at the start of the next cycle being slightly higher or lower than the horizontal velocity at the end of the current cycle. In our research, we adjust the initial angle based on the errors, as depicted in Figure 5. When the horizontal velocity error exceeds 0 or falls below 0, we utilize Equations (9) and (10) to decrease or increase the initial angle for the subsequent gait cycle with each correction limited to 5 degrees.

(9) $${\left(\theta \left(t_{cs}\right)\right)}_{{step}_{i+1}}={\left(\theta \left(t_{cs}\right)\right)}_{{step}_{i}}+F({Vel}_{err})$$

(10) $${Vel}_{err}={\left({Vel}_{h}\left(t_{cs}\right)\right)}_{{step}_{i+1}}-{\left({Vel}_{h}\left(t_{ce}\right)\right)}_{{step}_{i}}$$

where $F({Vel}_{err})$ is the calibration function that converts the drift velocity error at the end of this gait cycle into the correction angle for the initial sensor orientation angle (ISOA) of the next gait cycle.

### 2.8. Gait Parameters Estimation

#### 2.8.1. Stride Length

**Figure 6:**
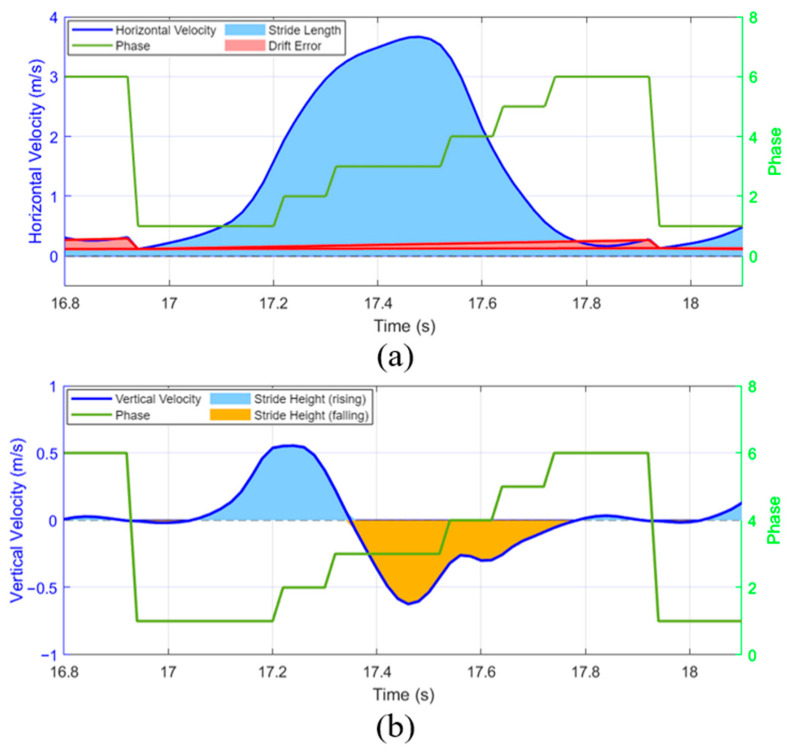
Spatial parameters estimation. (**a**) Stride length; (**b**) stride height.

The stride length (Figure 6) is the distance between two successive ground contacts made by the same foot. We integrate the horizontal velocity based on each gait cycle. A velocity drift correction is then performed at the end of each cycle [18].

(11) $$SL=\int_{t_{cs}}^{t_{ce}}{Vel}_{h}\left(\tau \right)d\tau -DE$$

(12) $$DE=\frac{{\left({Vel}_{h}\left(t_{ce}\right)\right)}_{{step}_{i}}-{\left({Vel}_{h}\left(t_{cs}\right)\right)}_{{step}_{i+1}}}{2}\cdot ST$$

We calculate the drift error (DE) using Equation (12), where ${\left({Vel}_{h}\left(t_{ce}\right)\right)}_{{step}_{i}}$ is the horizontal velocity at the end of the current gait cycle, ${\left({Vel}_{h}\left(t_{cs}\right)\right)}_{{step}_{i+1}}$ is the horizontal velocity at the start of the next gait cycle, and *ST* is the stride time of the current gait cycle.

#### 2.8.2. Stride Height

The definition of stride height (Figure 6) in this study is the maximum value of ankle displacement in the vertical direction relative to the ground during the gait cycle. We assume that the vertical position of the subject’s ankle at the start of the cycle is the same as that at the end. Therefore, stride height is the average of the positive and negative areas under the vertical velocity curve.

(13) $$SH=\frac{\int_{t_{cs}}^{t_{zc}}{Vel}_{v}\left(\tau \right)d\tau -\int_{t_{zc}}^{t_{ce}}{Vel}_{v}\left(\tau \right)d\tau }{2}$$

where $t_{zc}$ is the moment of the vertical velocity zero crossing from positive to negative.

#### 2.8.3. Stride Frequency

Stride frequency is the number of gait cycles that the same foot completes per second, with the unit being Hertz (Hz). It can be estimated by recording the period in each gait cycle and taking its reciprocal:

(14) $$SF=\frac{1}{t_{ce}-t_{cs}}$$

#### 2.8.4. Swing Ratio

Swing time is defined as the duration during which the foot is in the air [40,41], and it is considered to be the interval from Terminal-Contact (toe-off) to Initial-Contact (heel-strike) [15,20,24]. Therefore, the swing ratio is the percentage of the cycle during which the foot is in the air and does not touch the ground [9].

(15) $$SW=\frac{t_{IC}-t_{TC}}{t_{ce}-t_{cs}}*100\%$$

where $t_{IC}$ is the moment of IC, and $t_{TC}$ is the moment of TC.

### 2.9. Feature Derivation and Parameter Analysis

To enable clinically meaningful discrimination and reduce anthropometric confounding, the proposed evaluation framework analyzes four primary spatiotemporal parameters—stride length (SL), stride height (SH), stride frequency (SF), and swing ratio (SR)—and augments them with step-wise features that capture stability and symmetry. Although prior work often relies on per-subject means as summary descriptors, averages alone may be misleading: for example, stride length scales with body size/height and thus may obscure impairment severity when interpreted in isolation [15,21]. In addition, medical studies consistently report greater gait variability and asymmetry in patients with spinal disorders relative to healthy individuals, indicating that beat-to-beat fluctuations convey diagnostic value beyond central tendency [4,8].

Accordingly, for each primary gait parameter—stride length (SL), stride height (SH), stride frequency (SF), and swing ratio (SR)—three step-wise descriptors were computed across each walking trial: average (Avg), variation (Var), and variation ratio (VR). Avg characterizes the central tendency of gait performance, whereas Var quantifies local stride-to-stride fluctuations using consecutive absolute differences as a proxy for local gait instability. VR was designed as a normalized local variability descriptor that relates step-to-step fluctuation to the overall magnitude of the gait parameter, thereby reducing the influence of anthropometric scaling effects and facilitating inter-subject comparison.

These descriptors were computed separately for the left foot, right foot, and bilateral gait measurements, yielding a compact yet informative representation of (i) overall gait performance, (ii) stride-to-stride stability, and (iii) gait symmetry/asymmetry [42]. The resulting features were subsequently used for group comparison and as inputs to the composite scoring framework described in Section 2.9. The corresponding formulations are defined as follows:

(16) $$Avg=\frac{\sum_{n=1}^{N}x(n)}{N}$$

(17) $$Var=\frac{\sum_{n=2}^{N}\left|x\left(n\right)-x(n-1)\right|}{N-1}$$

(18) $$VR=\frac{\sum_{n=2}^{N}\left|x(n)-x(n-1)\right|}{\sum_{n=1}^{N}x(n)}*\frac{N}{N-1}$$

where *x*(*n*) denotes the gait parameter value (SL, SH, SF, or SR) at step n, and N represents the total number of steps acquired from the same foot during a walking task.

The proposed VR metric was designed to characterize normalized local gait variability. Specifically, the numerator evaluates cumulative consecutive step-to-step variation using N−1 step intervals, whereas the denominator represents cumulative gait magnitude across N gait samples. Accordingly, the factor N/(N−1) compensates for the mismatch in normalization bases between the two quantities and preserves consistency with the definitions of Avg and Var. From a statistical perspective, the resulting VR can be interpreted as a coefficient-of-variation-type normalized variability descriptor.

Physiologically, the normalization strategy reduces dependence on subject-specific anthropometric characteristics, such as body height and limb length. For example, stride length and its step-to-step variation are both expected to scale with participant height. By normalizing local variation with respect to the corresponding gait magnitude, the resulting VR becomes less sensitive to body-size-related effects and therefore improves inter-subject comparability. Because the factor N/(N−1) approaches unity for moderate-to-large sample sizes, its practical influence on the resulting VR values is limited; however, it was retained to preserve normalization consistency across walking trials with different step counts.

For each walking task, Avg, Var, and VR were computed for all four primary gait parameters, expanding the original four gait descriptors into twelve derived features. These features were further categorized according to left foot, right foot, and bilateral measurements to capture potential gait asymmetry. Consequently, a total of 36 gait-related features were obtained for each participant during each movement task.

### 2.10. Evaluation System

We developed a percentile-referenced composite scoring framework that maps 36 gait features (Avg/Var/VR across SL, SH, SF, SR for left, right, and both feet) to a single, interpretable index. For each feature, performance is first scaled against healthy controls (HC): scores of 100, 80, and 60 are anchored to the HC 75th, 50th, and 25th percentiles (PR75/PR50/PR25), respectively. The score range is 0–120. Values between 60 and 100 are obtained by linear interpolation within the HC interquartile range, whereas values > 100 to 120 (above PR75) and <60 down to 0 (below PR25) are assigned by linear extrapolation to preserve monotonicity and retain sensitivity in the tails. To ensure consistent clinical interpretation across all features, percentile-based scores were oriented such that higher scores always represent gait characteristics more similar to those of healthy controls. For features whose larger values indicate poorer gait performance, including variability-related descriptors (Var and VR), the percentile mapping was inverted prior to weighting and composite-score calculation. Next, we determine the weight of each feature based on the z-score of healthy subjects and patients’ results for each feature. The weight of each feature is calculated by dividing the Z-score of each feature by the total sum of the Z-scores of all 36 features. The weight of ${feature}_{i}$ is as follows:

(19) $${Weight}_{i}=\frac{{|\text{Z\_score}}_{i}|}{\sum_{j=1}^{36}{|\text{Z\_score}}_{j}|}$$

**Figure 7:**
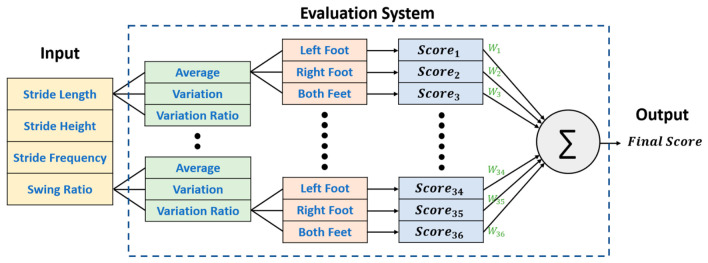
The architecture of the evaluation system.

The evaluation system, depicted in Figure 7, eventually multiplies the scores of all features by their respective weights and outputs the final score. The formula is shown as

(20) $$Final\;Score=\sum_{i=1}^{36}{Score}_{i}*{Weight}_{i}$$

## 3. Results

### 3.1. Composite Scoring Performance, Group Differences, and Validation Error

**Figure 8:**
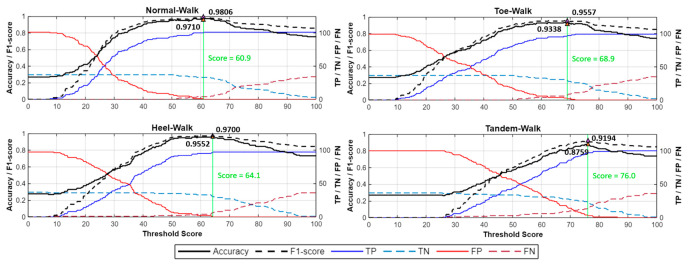
Classification performance of the proposed evaluation system under different threshold scores. The corresponding true positives (TPs), true negatives (TNs), false positives (FPs), false negatives (FNs), accuracy, and F1-score curves are shown for threshold-based classification across the four walking tasks. Triangles indicate the intersection points between the waveform and the green reference line, together with their corresponding values.

We conducted threshold-based classification using the percentile-referenced, separability-weighted composite score (Section 2.9). By systematically sweeping the decision threshold, the corresponding accuracy and F1-score curves were obtained as shown in Figure 8, enabling identification of the optimal classification threshold. The F1-score, which is widely adopted in event detection studies [34], provides a more informative assessment of misclassification than accuracy. The formulations of accuracy and F1-score are given as follows.

(21) $$Accuracy=\frac{TP+TN}{TP+TN+FP+FN}$$

(22) $$F1\;score=\frac{TP}{TP+\frac{FP+FN}{2}}$$

**Figure 9:**
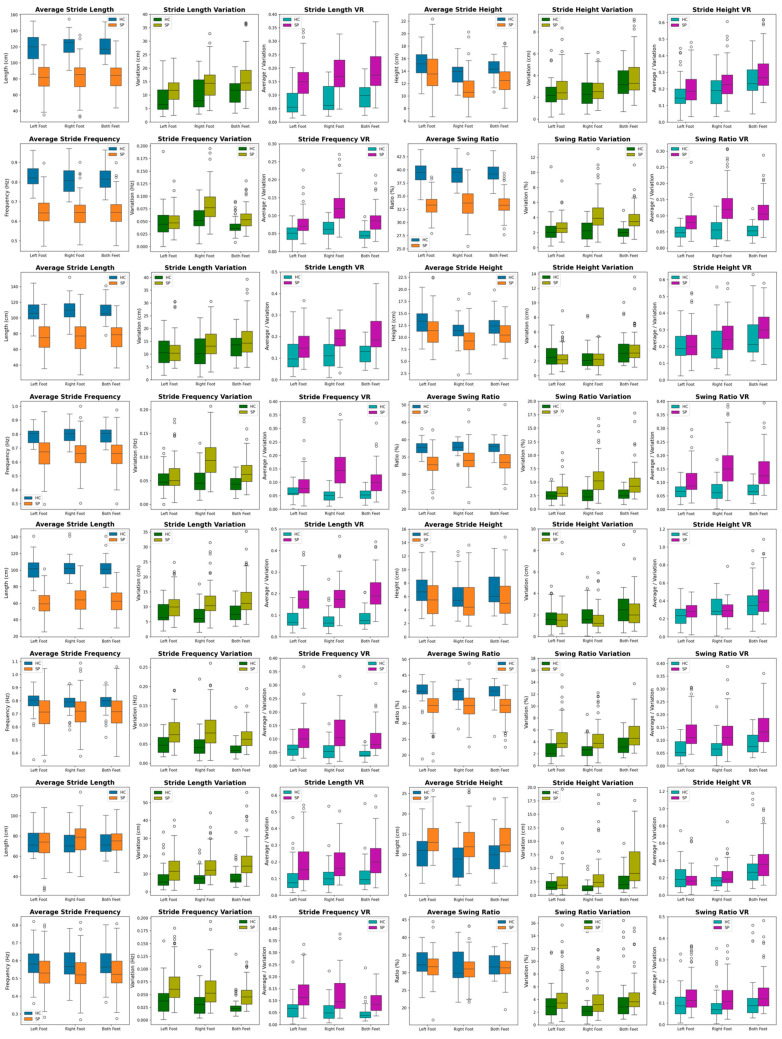
The boxplots of 36 features for all subjects in four gait movements.

Subsequently, for each walking task, the average (Avg), variation (Var), and variation ratio (VR) were computed for four primary gait parameters—stride length (SL), stride height (SH), stride frequency (SF), and swing ratio (SR)—for the left foot, right foot, and bilateral measurements. The distributions of these derived metrics were then compared between healthy controls (HC) and spinal patients (SP), as illustrated in Figure 9.

In the classification framework, TPs, TNs, FPs, and FNs denote true positives, true negatives, false positives, and false negatives, respectively, with all performance metrics normalized to the range [0, 1]. The optimal operating points were identified at thresholds of 60.9 (Regular-Walk), 68.9 (Toe-Walk), 64.1 (Heel-Walk), and 76.0 (Tandem-Walk), yielding corresponding accuracy/F1-scores of 0.9710/0.9806, 0.9338/0.9557, 0.9552/0.9700, and 0.8759/0.9194, respectively (Figure 9). These results demonstrate excellent discriminative performance for Regular-, Toe-, and Heel-Walk tasks, and comparatively robust performance for Tandem-Walk, despite its inherently constrained step geometry.

Parameter-level inferential statistics (Table 2) further clarify which metrics drive separability. At a significance level of α = 0.05, Regular-Walk showed HC–SP differences in SL (*p* = 0.005), SF (*p* = 0.009), and SR (*p* = 0.002), but not SH (*p* = 0.217). Toe-Walk likewise differed in SL (*p* = 0.011), SF (*p* = 0.027), and SR (*p* = 0.048), with SH (*p* = 0.440) being non-significant. In Heel-Walk, only SL reached significance, whereas SF, SR, and SH did not. Tandem-Walk yielded no significant differences across the four parameters. Collectively, these findings identify SL, SF, and SR as the most discriminative parameters in Regular- and Toe-Walk, with SL retaining value in Heel-Walk and limited separability in Tandem-Walk, consistent with its task constraint.

The comparatively lower classification performance observed in Tandem-Walk likely reflects both task-specific biomechanical constraints and, to a lesser extent, sensing-related limitations associated with narrow-base cross-step movements. From a clinical perspective, Tandem-Walk imposes strict heel-to-toe alignment and constrained step geometry, thereby reducing natural stride-length differences between healthy controls and spinal patients. In addition, Tandem-Walk introduces more complex lower-limb kinematics, including partial foot crossover and prolonged double-support phases, which may increase the difficulty of gait-event segmentation and parameter estimation using ankle-mounted inertial sensors.

Nevertheless, variability-related descriptors remained relatively informative in this condition, suggesting that the proposed sensing framework retained sensitivity to instability and asymmetry despite reduced discrimination in average gait parameters. Collectively, these findings identify SL, SF, and SR as the most discriminative parameters in Regular- and Toe-Walk, with SL retaining a discriminative value in Heel-Walk and variability-related descriptors contributing relatively greater importance in Tandem-Walk under constrained gait conditions.

Algorithmic performance was validated against the Vicon motion-capture system using the Group I validation cohort. The corresponding validation dataset comprised 1019 gait steps collected from 15 healthy participants across the four walking tasks (Table 2). The reported step count represents event-level gait samples used for algorithm validation rather than the number of study participants. For each gait parameter and walking condition, estimation accuracy was quantified using the mean ± standard deviation of the estimation error, together with mean absolute error (MAE) and Root Mean Square Error (RMSE), defined as follows:

(23) $$MAE=\frac{\sum_{n=1}^{N}\left|x(n)\;-\;m(n)\right|}{N}$$

(24) $$RMSE=\sqrt{\frac{\sum_{n=1}^{N}{\left[x(n)-m(n)\right]}^{2}}{N}}$$

where x(n) denotes the gait parameter estimated by the proposed algorithm and m(n) represents the corresponding Vicon reference measurement. Across all walking tasks, the proposed framework achieved MAE ranges of 4.877–7.863 cm for stride length (SL), 1.766–2.417 cm for stride height (SH), 0.009–0.027 Hz for stride frequency (SF), and 1.588–1.843% for swing ratio (SR). These results indicate centimeter-level spatial estimation accuracy and low temporal estimation error under diverse gait conditions, demonstrating the capability of the proposed ankle-mounted IMU framework to provide stable and reliable quantification of clinically relevant gait parameters.

Importantly, the validation experiments were conducted using synchronized Vicon measurements as the reference standard, whereas the proposed framework itself relies solely on wearable ankle-mounted IMUs during routine operation. The low estimation errors observed across all walking tasks therefore support the feasibility of the proposed system for real-time gait assessment without requiring continuous optical motion-capture infrastructure.

Furthermore, the concordance between low parameter estimation error and strong composite-score discrimination suggests that the proposed framework can effectively capture clinically meaningful gait alterations associated with degenerative spinal disorders. These findings support the potential utility of the system for functional gait assessment and longitudinal monitoring in clinical and point-of-care settings.

### 3.2. Smart Phone Application Implementation

We developed a companion Android application (C++/Java, Android Studio) to execute the proposed pipeline on-device in real time. After Bluetooth discovery and secure pairing with the two ankle IMUs, the app streams tri-axial acceleration and angular velocity at 50 Hz (configured to ±16 g and ±1000 deg/s) and performs causal filtering, finite-state-machine gait-event detection, orientation/velocity updates, and feature derivation directly on the phone. A task selector launches Regular-, Toe-, Heel-, or Tandem-Walk; during acquisition the interface displays per-step stride length (SL), stride height (SH), stride frequency (SF), and swing ratio (SR) for left/right feet together with running indicators of total gait duration and average velocity. Upon task completion (e.g., pre-specified step count reached or operator returning to the main panel), the application summarizes per-parameter averages and the percentile-referenced, separability-weighted composite score (§2.9) and offers one-tap save/export of raw and processed data. Online quality controls—timestamp alignment, detection of >100 ms streaming gaps, range checks, and baseline inconsistencies suggestive of strap slippage—prompt immediate repetition when necessary to safeguard downstream validity.

**Figure 10:**
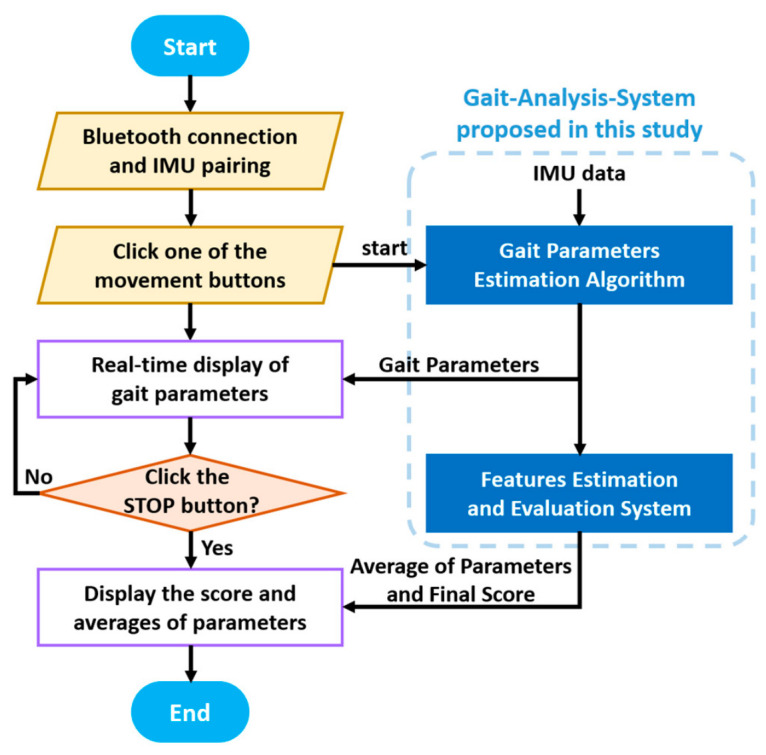
Mobile application workflow with integrated on-device inference. After Bluetooth pairing, dual-ankle IMU streams (50 Hz) feed the Gait Parameters Estimation Algorithm (SL/SH/SF/SR), whose outputs enter the Features Estimation and Evaluation System to compute Avg/Var/VR and the composite score; live parameters are displayed during acquisition, followed by a summary view at task completion.

**Figure 11:**
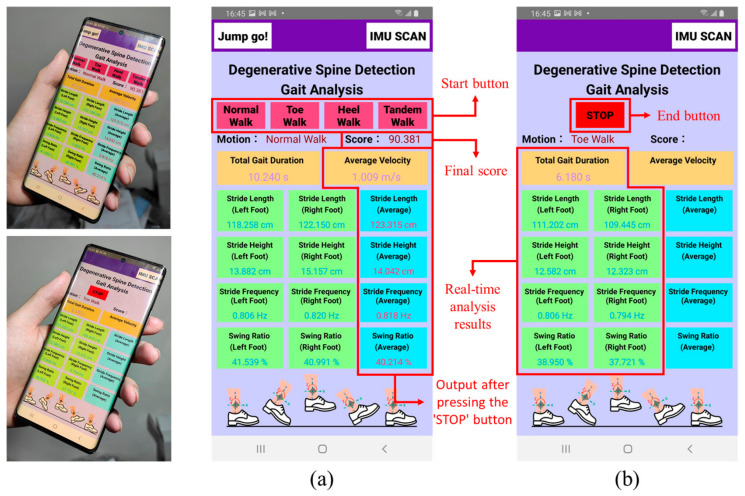
Main activity screens. (**a**) Post-task summary: final composite score, total gait duration, average velocity, and per-parameter (left/right/average) results with save/export options. (**b**) During-task display: real-time SL/SH/SF/SR for both feet with a task header and live indicators to facilitate protocol adherence and quality control. The application operates solely using IMU measurements during routine deployment and does not require optical cameras.

The software/data path and UI logic are summarized in Figure 10 and Figure 11, respectively. Following validation against the Vicon reference system, the proposed framework was implemented as a standalone mobile application for real-time gait assessment. During routine operation, only the two ankle-mounted IMUs and Bluetooth communication are required; no optical cameras or motion-capture equipment are involved. The app was exercised both in the motion-capture laboratory and in clinical corridors during outpatient visits. In the laboratory, real-time readouts enabled rapid verification of sensor synchronization and axis alignment prior to Vicon trials, reducing setup iterations. In clinical use, clinicians reported that instantaneous feedback supported protocol adherence (step counts and task order), enabled bedside quality control (early identification of streaming gaps or attachment issues), and provided a quick impression of stability and asymmetry via Var/VR trends without waiting for offline analysis. The on-phone inference remained responsive throughout all tasks, and the composite score furnished a single, interpretable index that fit clinic workflow for triage, progress reviews, and post-visit documentation [43,44].

## 4. Discussion

This work presents an IMU-based framework to quantify gait alterations associated with lumbar spine disorders and to summarize performance via a composite score. The study comprised two components: (i) a real-time algorithm that estimates stride length (SL), stride height (SH), stride frequency (SF), and swing ratio (SR) from tri-axial acceleration and angular velocity; and (ii) a feature-to-score mapping that aggregates averages, step-to-step variability, and asymmetry into a percentile-referenced, separability-weighted index. While SL, SF, and SR have been routinely derived from wearable sensors for clinical evaluation [21,32,34,41,45], our implementation extends these estimators across four distinct tasks (Regular-, Toe-, Heel-, and Tandem-Walk), enabling task-specific probing within a unified pipeline. Using Vicon ground truth, the SL error (RMSE%) ranged from 6.3% to 9.0%, which falls within the spread reported by prior studies (1.9–9.3%) [18,21,29,32,34,45]. Therefore, the proposed framework demonstrates estimation accuracy comparable to existing approaches while providing the additional advantage of multi-task gait assessment within a single wearable platform. However, it should be noted that the Vicon-based validation was performed exclusively in Group I, which consisted of healthy young adults. Although the proposed framework demonstrated favorable agreement with the motion-capture reference system under controlled laboratory conditions, gait characteristics in patients with degenerative spinal disorders may differ substantially from those of healthy individuals. Therefore, the reported estimation accuracy may not fully reflect performance in the target patient population, and future studies incorporating motion-capture validation in patient cohorts will be valuable for further assessing algorithm robustness across diverse gait impairments.

Group comparisons reinforce known disease signatures while clarifying task effects. In Regular- and Toe-Walk, SL, SF, and SR differed significantly between healthy controls and spinal patients (all *p* < 0.05), whereas SH did not; in Heel-Walk, only SL was significant; and in Tandem-Walk, none of the four parameters differed significantly. The absence of SL differences in Tandem-Walk is consistent with the task’s geometric constraint—requiring heel-to-toe progression during double support—yielding strides approximating twice the foot length and thereby masking pathology-related shortening. These findings align with reports that lumbar spine disorders are characterized by reduced cadence/stride length and prolonged temporal phases [4,6,7,12].

At the feature level (Figure 9), spinal patients consistently exhibit greater step-to-step variability and asymmetry than healthy controls. Specifically, among single-foot descriptors, 28 of 32 variation (Var) features and 30 of 32 variation-ratio (VR) features are larger in patients; for both-feet aggregates, 15 of 16 Var features and all VR features are larger in patients. These observations are consistent with prior studies reporting increased gait instability and asymmetry in spinal disorders [8,46]. Because feature weights in the proposed scoring framework reflect between-group separability, descriptors exhibiting larger differences between healthy controls and spinal patients naturally receive higher weights. After weighting, mean-value descriptors contributed most strongly to the final composite score in Regular-, Toe-, and Heel-Walk, followed by VR and then Var descriptors. In contrast, VR-related features became relatively more informative in Tandem-Walk, consistent with the geometric constraints of this task, which reduce the discriminative value of mean gait parameters. Overall, the most discriminative parameter–task combinations were stride length (SL), stride frequency (SF), and swing ratio (SR) during Regular-Walk and Toe-Walk, together with stride length (SL) during Heel-Walk. These features consistently exhibited the largest separations between healthy controls and spinal patients. In contrast, Tandem-Walk showed comparatively limited discriminative ability across most feature categories.

Threshold-based classification further supports these observations. Decision thresholds of 60.9, 68.9, 64.1, and 76.0 for Regular-, Toe-, Heel-, and Tandem-Walk yielded accuracies of 0.9710, 0.9338, 0.9552, and 0.8759 and corresponding F1-scores of 0.9806, 0.9557, 0.9700, and 0.9194, respectively. In the Tandem-Walk condition, however, the reduced separation between healthy-control and patient distributions increased the likelihood of misclassification; for example, 14 of 37 healthy participants were classified as patients at the optimal threshold. Overall, Regular-, Toe-, and Heel-Walk provided substantially stronger discrimination between healthy controls and spinal patients, whereas Tandem-Walk appeared less suitable as a primary screening task because of its inherent biomechanical constraints. Nevertheless, Tandem-Walk may still provide complementary information regarding gait instability, balance control, and asymmetry, particularly through variability-related descriptors (Var and VR), and therefore may be useful as a secondary assessment task within a comprehensive gait evaluation framework. In addition, Tandem-Walk involves more complex three-dimensional movement patterns, including narrow-base progression, balance-correction maneuvers, and partial foot crossover. These characteristics may reduce the validity of the planar-motion approximation adopted in the present framework and partially contribute to the lower discrimination performance observed in this task. Future studies may incorporate full three-dimensional kinematic modeling or advanced sensor-fusion approaches to further improve performance under such conditions [47].

Despite these encouraging findings, several limitations should be acknowledged. First, the study cohort exhibited an imbalance between healthy controls and spinal patients, with substantially more patient recordings than healthy recordings. This distribution reflects the actual clinical prevalence and recruitment characteristics of degenerative spinal disorders encountered in routine clinical practice. Consequently, no resampling, oversampling, or class-balancing strategy was applied during the analysis. Although the proposed composite scoring framework demonstrated robust classification performance despite this imbalance, future studies involving larger and more balanced cohorts would further strengthen the generalizability and statistical robustness of the proposed framework. Furthermore, all participants in the present study were recruited from a single medical center. Although the proposed system demonstrated promising performance under real-world clinical conditions, independent validation using multicenter cohorts will be necessary to evaluate reproducibility, robustness, and generalizability across different clinical environments, patient populations, and deployment settings.

In addition, the current implementation employs a fixed ankle-height approximation to simplify real-time deployment and minimize subject-specific calibration requirements. Although the resulting influence on stride-length estimation is expected to be relatively small compared with other sources of estimation uncertainty, such as gait-event detection and velocity estimation, future studies may incorporate subject-specific anthropometric calibration to further improve estimation accuracy and personalization. Moreover, the reported classification performance should be interpreted in the context of the current study design, where feature weighting and threshold selection were derived from the same cohort. Although the proposed framework demonstrated promising discrimination between healthy controls and spinal patients, future studies incorporating independent validation datasets, cross-validation procedures, and multicenter cohorts will be important for further evaluating robustness and generalizability.

Second, a noticeable age difference existed between the healthy control group and the spinal patient group. Because gait characteristics are known to change with aging, part of the observed differences in spatiotemporal gait parameters may have been influenced not only by disease-related impairments but also by age-related physiological changes. While the primary objective of this study was to develop and validate an IMU-based gait assessment framework under realistic clinical conditions, future investigations should recruit age-matched healthy controls to better isolate the specific effects of degenerative spinal disorders on gait performance. Age-matched validation cohorts would also enable a more rigorous assessment of the disease-specific discriminative capability of the proposed framework. Accordingly, the present findings should be interpreted as reflecting the combined effects of spinal pathology under real-world clinical recruitment conditions rather than a strictly age-controlled comparison. In addition, stride-to-stride variability was estimated from relatively short walking trials consisting of approximately 6–10 gait cycles. Although these measures were sufficient for the screening-oriented objectives of the present study, future investigations employing longer-duration recordings may further improve the statistical stability and clinical representativeness of variability-related descriptors.

Third, the present study focused primarily on functional gait assessment rather than disease-specific stratification. Accordingly, the proposed classification framework was designed to distinguish healthy gait from gait affected by degenerative spinal disorders in a clinically practical manner, rather than to differentiate among individual spinal diagnoses. The spinal patient cohort included heterogeneous diagnoses, such as low back pain, lumbar disk herniation, and lumbar spinal stenosis, which may exhibit distinct biomechanical characteristics and disease progression patterns. Future studies incorporating larger disease-specific cohorts, age-matched controls, and longitudinal follow-up data may further clarify the relationships among gait abnormalities, disease severity, treatment response, and clinical outcomes.

Despite these limitations, the proposed framework demonstrated consistent gait-parameter estimation accuracy, robust discrimination between healthy controls and spinal patients, and practical feasibility for real-time deployment using only two ankle-mounted IMUs. These findings support the potential of the proposed system as a scalable, interpretable, and clinically deployable tool for point-of-care gait assessment and longitudinal monitoring of degenerative spinal disorders.

## 5. Conclusions

We presented a real-time, IMU-based framework for detecting gait impairments associated with degenerative spinal disease, integrating (i) an on-device algorithm that estimates stride length, stride height, stride frequency, and swing ratio from two ankle-mounted IMUs, and (ii) a percentile-referenced, separability-weighted composite score that converts multi-parameter gait signatures into a single interpretable index. Across participants performing Regular-, Toe-, Heel-, and Tandem-Walk tasks, the proposed evaluation system achieved classification accuracies of 97.10%, 93.38%, 95.52%, and 87.59%, respectively. Consistent with characteristics of spinal pathology, the analysis revealed that stride length, stride frequency, and swing ratio were significantly reduced in patients compared with healthy controls during Regular-, Toe-, and Heel-Walk. In addition, gait variability and asymmetry were consistently elevated in the patient group across all four walking tasks.

This work advances the state of the art by (i) delivering edge-enabled, real-time inference from only two low-power IMUs, thereby reducing hardware complexity, cost, and latency while preserving clinically relevant accuracy; (ii) supporting multi-task assessment within a unified pipeline so that spinal deficits can be probed under different biomechanical demands rather than a single walking condition; (iii) augmenting conventional averages with step-to-step variability and asymmetry features and aggregating them through a transparent composite score suited to threshold-based screening and longitudinal monitoring; and (iv) emphasizing deployability via standardized ankle placement, 50 Hz sampling, and lightweight computation that fit bedside and corridor workflows without specialized infrastructure. Together, these properties bridge laboratory-grade quantification and scalable point-of-care assessment, offering a practical path for routine triage, progress tracking, and treatment evaluation in clinical settings.

## Figures and Tables

**Table 1:** (**a**) The classification and quantity of recorded data from IMU. (**b**) Demographic and clinical characteristics of study participants.

(a)							
Database	Measurement Location	Subjects	Record with Vicon	Number of Samples			
				Regular-Walk	Toe-Walk	Heel-Walk	Tandem-Walk
Group I	Laboratory	Healthy	Yes	36	29	27	27
Group II	School corridor	Healthy	No	37	37	37	37
Group III	Clinic	Patient	No	101	99	97	100
(**b**)							
Variable		Group I Validation Cohort		Group II Healthy Controls		Group III Spinal Patients	
Purpose		Algorithm validation		Healthy reference cohort		Clinical evaluation cohort	
Number of participants, *n*		15		37		101	
Age (years)		27.5 ± 9.6		28.5 ± 8.4		60.1 ± 14.1	
Sex (male/female)		10/5		27/10		35/66	
Height (cm)		169.5 ± 4.9		169.9 ± 5.0		160.8 ± 9.4	
Weight (kg)		66.4 ± 7.2		66.4 ± 8.0		66.1 ± 13.9	
BMI (kg/m^2^)		23.6 ± 2.0		23.0 ± 2.0		25.4 ± 4.5	
Clinical diagnosis		Healthy		Healthy		Patient	
Low back pain		–		–		85 (84.16%)	
Sciatica		–		–		72 (71.29%)	
Claudication		–		–		34 (33.66%)	
Weakness		–		–		14 (13.86%)	
Numbness		–		–		70 (69.3%)	
C-spine with unsteady gait		–		–		2 (1.98%)	

The data for each motion includes 6 to 10 steps.

**Table 2:** Statistical results of gait parameter estimation errors and the simulation results of clinical data.

Motion	Parameter (Unit)	Error Analysis				Clinical Data Analysis		
		HC ^a^ (Mean ± SD)	Vicon (Mean ± SD)	MAE	RMSE	HC ^b^ (Mean ± SD)	Patient (Mean ± SD)	*p*-Value
Regular Walking	SL (cm)	117.52 ± 22.09	120.71 ± 24.34	7.863	9.774	119.99 ± 13.05	83.02 ± 15.78	0.005
	SH (cm)	13.49 ± 3.57	12.93 ± 2.93	2.087	2.783	14.37 ± 1.40	12.64 ± 2.31	0.217
	SF (Hz)	0.78 ± 0.09	0.79 ± 0.08	0.027	0.038	0.82 ± 0.07	0.65 ± 0.07	0.009
	SR (%)	38.62 ± 2.89	38.44 ± 2.00	1.777	2.280	39.40 ± 1.95	33.42 ± 2.11	0.002
Toe Walking	SL (cm)	110.35 ± 12.47	114.21 ± 12.49	5.930	7.139	109.36 ± 13.13	75.80 ± 18.33	0.011
	SH (cm)	11.70 ± 3.06	10.84 ± 1.89	2.306	3.191	12.45 ± 2.23	10.73 ± 2.56	0.440
	SF (Hz)	0.80 ± 0.07	0.80 ± 0.07	0.025	0.035	0.80 ± 0.06	0.66 ± 0.12	0.027
	SR (%)	37.03 ± 2.45	35.55 ± 2.57	1.843	2.455	37.80 ± 1.69	34.45 ± 5.09	0.048
Heel Walking	SL (cm)	100.00 ± 12.39	103.83 ± 12.55	5.476	6.876	102.22 ± 13.00	62.12 ± 16.07	0.002
	SH (cm)	6.10 ± 2.55	5.37 ± 1.79	1.766	2.276	6.27 ± 2.50	5.87 ± 2.91	0.693
	SF (Hz)	0.78 ± 0.07	0.78 ± 0.07	0.018	0.023	0.78 ± 0.08	0.72 ± 0.14	0.432
	SR (%)	40.88 ± 3.38	41.02 ± 2.61	1.588	2.183	39.50 ± 3.10	35.15 ± 3.72	0.160
Tandem Walking	SL (cm)	66.95 ± 8.76	68.60 ± 6.20	4.877	6.185	73.46 ± 11.71	74.40 ± 13.42	0.936
	SH (cm)	8.28 ± 4.86	8.13 ± 2.50	2.417	3.398	9.88 ± 4.76	15.09 ± 9.57	0.274
	SF (Hz)	0.58 ± 0.07	0.58 ± 0.08	0.009	0.015	0.58 ± 0.10	0.53 ± 0.10	0.607
	SR (%)	34.37 ± 3.11	35.03 ± 3.46	1.773	2.148	32.09 ± 2.97	31.25 ± 3.25	0.777

^a^ The healthy control was recorded in the laboratory (Group I). Due to spatial constraints, each movement was repeated for 5 to 10 steps. ^b^ The healthy control was recorded in the school corridor (Group II), with each movement repeated for 10 steps.

## Data Availability

The data presented in this study are not publicly available due to privacy and ethical restrictions. Access to the data requires approval from the Health and Welfare Data Science Center (HWDC), Ministry of Health and Welfare, Taiwan.

## Abbreviations

The following abbreviations are used in this manuscript:

IMUs	Inertial Measurement Units
DSD	Degenerative lumbar spine disease
ZUPT	Zero-velocity update
LDH	Lumbar disk herniation
LSS	Lumbar spinal stenosis
SL	Stride length
SH	Stride height
SF	Stride frequency
SR	Swing ratio
